# Emotional Dysregulation, Impulsivity, and Interoceptive Awareness in Individuals with Alcohol Use Disorder

**DOI:** 10.1192/j.eurpsy.2025.640

**Published:** 2025-08-26

**Authors:** F. Seven, M. B. Sönmez

**Affiliations:** 1 Psychiatry, Edirne Uzunköprü State Hospital; 2 Psychiatry, Trakya University School of Medicine, Edirne, Türkiye

## Abstract

**Introduction:**

Impairment in emotion regulation and impulsivity are components of addiction-related mechanisms. The ability to perceive the internal state of the body is known as interoceptive awareness (IA). Impaired IA is believed to contribute to the development and progression of alcohol use disorder (AUD). IA is considered to have two dimensions: interoceptive accuracy (IAc), which measures precise monitoring of bodily sensations, and interoceptive sensibility (IS), which reflects the subjective experience of these sensations. Traits associated with alcohol use vulnerability, such as emotional dysregulation and impulsivity, may also be linked to IA.

**Objectives:**

Our objective was to compare emotional dysregulation, impulsivity, IAc, and IS levels between abstinent patients with AUD and healthy controls. Additionally, we aimed to investigate potential associations between the dimensions of IA and emotional dysregulation and impulsivity.

**Methods:**

The study included 52 abstinent patients with AUD and 52 healthy control subjects. Of the participants, 92.3% (n=48) in each group were male, and 7.7% (n=4) were female. Emotional dysregulation was assessed using the 16-item Difficulties in Emotion Regulation Scale (DERS-16), and impulsivity was measured using the Barratt Impulsiveness Scale 11 (BIS-11). IAc was evaluated using the heart rate tracking task, which assessed participants’ awareness of their own heartbeat by comparing the number of heartbeats they perceived with an objective heart rate measurement. IS was measured using the Multidimensional Assessment of Interoceptive Awareness Version 2 (MAIA-2). The study included patients who had completed detoxification and been abstinent for at least three weeks while participating in or undergoing a 28-day abstinence-based inpatient treatment program.

**Results:**

Individuals with AUD scored significantly higher on self-reported measures of emotional dysregulation (AUD group: 41.50 ± 17.66; control group: 31.19 ± 8.93; p < 0.001, F = 14.106) and impulsivity (AUD group: 61.63 ± 12.30; control group: 53.06 ± 7.50; p < 0.001, F = 17.828), and significantly lower on the heart rate tracking task (IAc) (AUD group: 0.65 ± 0.15; control group: 0.84 ± 0.13; p < 0.001, F = 43.615). No significant difference was found in self-reported IS scores (AUD group: 114.06 ± 21.38; control group: 113.37 ± 13.52; p = 0.844, F = 0.039). There was a significant correlation between emotion dysregulation and impulsivity scores (r = 0.633, p < 0.001). IAc and IS scores showed significant negative correlations with emotional dysregulation scores (r = -0.243, p = 0.013; r = -0.425, p < 0.001, respectively) and impulsivity scores (r = -0.204, p = 0.038; r = -0.416, p < 0.001, respectively).

**Conclusions:**

Our findings support the hypothesis that emotional dysregulation and impulsivity, which are linked to the development and progression of AUD, are associated with interoceptive processes.

**Disclosure of Interest:**

None Declared

